# Relationship between hypothyroidism and periodontitis: A scoping review

**DOI:** 10.1002/cre2.247

**Published:** 2019-09-26

**Authors:** Hajer A. Aldulaijan, Robert E. Cohen, Elizabeth M. Stellrecht, Michael J. Levine, Lisa M. Yerke

**Affiliations:** ^1^ Department of Periodontics and Endodontics University at Buffalo, State University of New York, School of Dental Medicine Buffalo New York; ^2^ Present address: Department of Periodontics and Community Dentistry King Saud University, College of Dentistry Riyadh Saudi Arabia

**Keywords:** hypothyroidism, periodontitis, scoping review

## Abstract

**Aim:**

The objective of this study was to assess the existing literature to determine if a relationship exists between hypothyroidism and periodontitis.

**Methods:**

We used a modified approach to the Preferred Reporting Items for Systematic Reviews and Meta‐Analyses by searching five databases in addition to the gray literature. Keywords in the title and abstract fields, as well as subject headings for both periodontal disease and hypothyroidism, were used to search the existing literature for publications relevant to evaluation of the thyroid–periodontitis relationship.

**Results:**

The authors screened 847 unique publications which, after applying inclusion and exclusion criteria, yielded 29 publications, which were further analyzed for relevance and applicability. Most of the included papers were cross‐sectional studies and retrospective chart reviews. Following critical analysis, four publications, including one abstract, were used to further assess the hypothyroid–periodontitis relationship.

**Conclusions:**

There are very few high‐quality studies describing the potential association between hypothyroidism and periodontitis. In general, and among the included papers with the fewest confounding factors, a positive relationship between hypothyroidism and periodontitis was found. Further well‐controlled, prospective clinical and immunologic studies will be required to confirm that relationship.

## 1 INTRODUCTION

Relationships between periodontal diseases and a variety of systemic conditions have been described in the literature, including cardiovascular disease, cerebrovascular disease, respiratory disease, pregnancies resulting in preterm delivery or low birth weight infants, diabetes mellitus, rheumatoid arthritis, obesity, osteoporosis, and stress (R. Genco, Offenbacher, & Beck, 2002; R. J. Genco & Borgnakke, 2013). In addition, there is evidence that the severity and prevalence of periodontitis is greater in individuals with autoimmune diseases such as rheumatoid arthritis (Wolff et al., 2014) and inflammatory bowel diseases (Brito et al., 2008; Vavricka et al., 2013). The association between those conditions and periodontal disease might be due to common immunoinflammatory pathways in disease pathogenesis (Araujo, Melo, & Lima, 2015). It has been hypothesized that inflammatory mediators such as prostaglandin E_2_, interleukin‐1β, tumor necrosis factor‐α, and matrix metalloproteinases released locally as a consequence of periodontal disease lead to an alteration in bone hemostasis and might represent a risk factor for other systemic diseases such as rheumatoid arthritis (Araujo et al., 2015; Habashneh, Khader, Alhumouz, Jadallah, & Ajlouni, 2012). It is well established that the endocrine system can modulate the immune system in a bidirectional manner (Klein, 2006). Indeed, the relationship between diabetes mellitus and periodontitis offers a potential explanation for how acute and chronic inflammation, such as what occurs during periodontitis, might affect thyroid hormone production (R. J. Genco & Borgnakke, 2013).

Hypothyroidism is the most common hormone abnormality in humans and represents the most common thyroid disease. Hypothyroidism presents with a wide range of severity, from asymptomatic individuals to patients with multisystem failure (Roberts & Ladenson, 2004) due to decreased production of thyroxine (T_4_), triiodothyronine (T_3_), and calcitonin (Little, 2006), which causes decreased bone metabolism, maturation, and turnover and negatively affects bone homeostasis (Mosekilde & Melsen, 1978).

Consequently, the primary aim of this scoping review was to evaluate the quality and quantity of the existing literature describing the potential relationship between hypothyroidism and periodontitis. A secondary aim was to assess whether a relationship exists between hypothyroidism and periodontal disease severity.

## 2 MATERIALS AND METHODS

This review used the Preferred Reporting Items for Systematic Reviews and Meta‐Analyses reporting guideline (Moher, Liberati, Tetzlaff, & Altman, 2010) and modified them to apply to this scoping review.

An initial electronic search was created by a health sciences librarian and completed on September 4, 2018, and supplemented on April 26, 2019, in the following databases: PubMed, Embase via http://Embase.com, CINAHL via EBSCOhost, Web of Science, and Evidence‐Based Medicine Reviews via Ovid, a database composed of seven other databases (including Cochrane Database of Systematic Reviews, Cochrane Register of Controlled Trials, ACP Journal Club, Health Technology Assessment, Database of Abstracts of Reviews of Effects, Cochrane Methodology Register, and National Health Service Economic Evaluation Database). The search included keywords for both hypothyroidism and periodontal disease and included subject terms from each database's thesaurus (such as Medical Subject Headings or MeSH) when possible, as well as keyword searching of title and abstract fields. No date or language restrictions were applied to the initial search. The exact search terminology used in PubMed can be found in Table 1. That search was applied to each database, with MeSH terms translated to reflect the controlled vocabulary of each individual database.

**Table 1:** Search strategy for PubMed

1. “periodontal diseases”[MeSH Terms]
2. periodont*[Title/Abstract]
3. gingiv*[Title/Abstract]
4. peri implantitis[Title/Abstract]
5. alveolar bone[Title/Abstract]
6. ((((“periodontal diseases”[MeSH Terms]) OR periodont*[Title/Abstract]) OR gingiv*[Title/Abstract]) OR peri implantitis[Title/Abstract]) OR alveolar bone[Title/Abstract]
7. “thyroid diseases”[MeSH Terms]
8. thyroid*[Title/Abstract]
9. hypothyroid*[Title/Abstract]
10. ((“thyroid diseases”[MeSH Terms]) OR thyroid*[Title/Abstract]) OR hypothyroid*[Title/Abstract]
11. ((((((((((“periodontal diseases”[MeSH Terms]) OR periodont*[Title/Abstract]) R gingiv*[Title/Abstract]) OR peri implantitis[Title/Abstract]) OR alveolar bone[Title/Abstract])) AND (((“thyroid diseases”[MeSH Terms]) OR thyroid*[Title/Abstract]) OR hypothyroid*[Title/Abstract])

To assess the gray literature, the following resources were searched: Proquest Dissertations and Theses Global, http://clinicaltrials.gov, International Association for Dental Research (IADR) Abstract Archive (2001–present), American Thyroid Association (ATA) Meeting Abstracts (2011–present), and OpenGrey.eu. Proquest Dissertations and Theses Global allows for complex Boolean searching, which enabled the authors to use the same search that was used in the previously mentioned nongray databases. However, a search using the terms “periodontal disease” and “thyroid” or “periodontal disease” and “hypothyroidism” were used for searching the other gray literature resources. No date or language restrictions were used at this search stage with the exceptions of the IADR Abstract Archives and the ATA Meeting Abstracts, as the on‐line IADR Abstract Archives begins in the year of 2001, and the ATA Meeting Abstracts is freely available beginning only in 2011.

Our initial search on September 4, 2018, yielded 1,182 articles. When the search was updated on April 26, 2019, an additional 36 unique articles were identified. Three hundred seventy‐one articles were then excluded due to duplication. Collectively, both searches resulted in 847 articles that were subsequently screened by two reviewers (H. A. amd L. Y.) and resulted in the exclusion of 746 articles due to irrelevant title and/or abstract (i.e., not related to periodontitis and hypothyroidism). Our inclusion and exclusion criteria are summarized in Table 2.

**Table 2:** Aim 1: Inclusion and exclusion criteria

Inclusion criteria
1. Full‐text available.
2. Both human and animal studies.
3. All study designs.
4. True periodontal outcome = any measure of periodontal status (bone loss, pocket depth, missing teeth, need for surgery, bleeding, suppuration, radiographic evidence of bone loss, including teeth or implants, or previous diagnosis of periodontal disease).
5. Presence of hypothyroidism confirmed by laboratory testing (thyroid panel—TSH, free T_4_, free T_3_, or total T_3_) or medication.
Exclusion criteria
1. Nonmammalian studies.
2. Languages other than English.
3. Hyperthyroidism or thyroid cancer.
4. Wrong comparator = no comparison between periodontitis and hypothyroidism.
5. Wrong intervention = the intervention was not for periodontitis diagnosis.
6. Irrelevant outcome = the outcome wasn't related to periodontitis/hypothyroidism.
7. Wrong patient population = no comparison of periodontitis and/or hypothyroidism.
8. Journals of publication not cited in open access checklist for predatory publishers.

**Figure 1:**
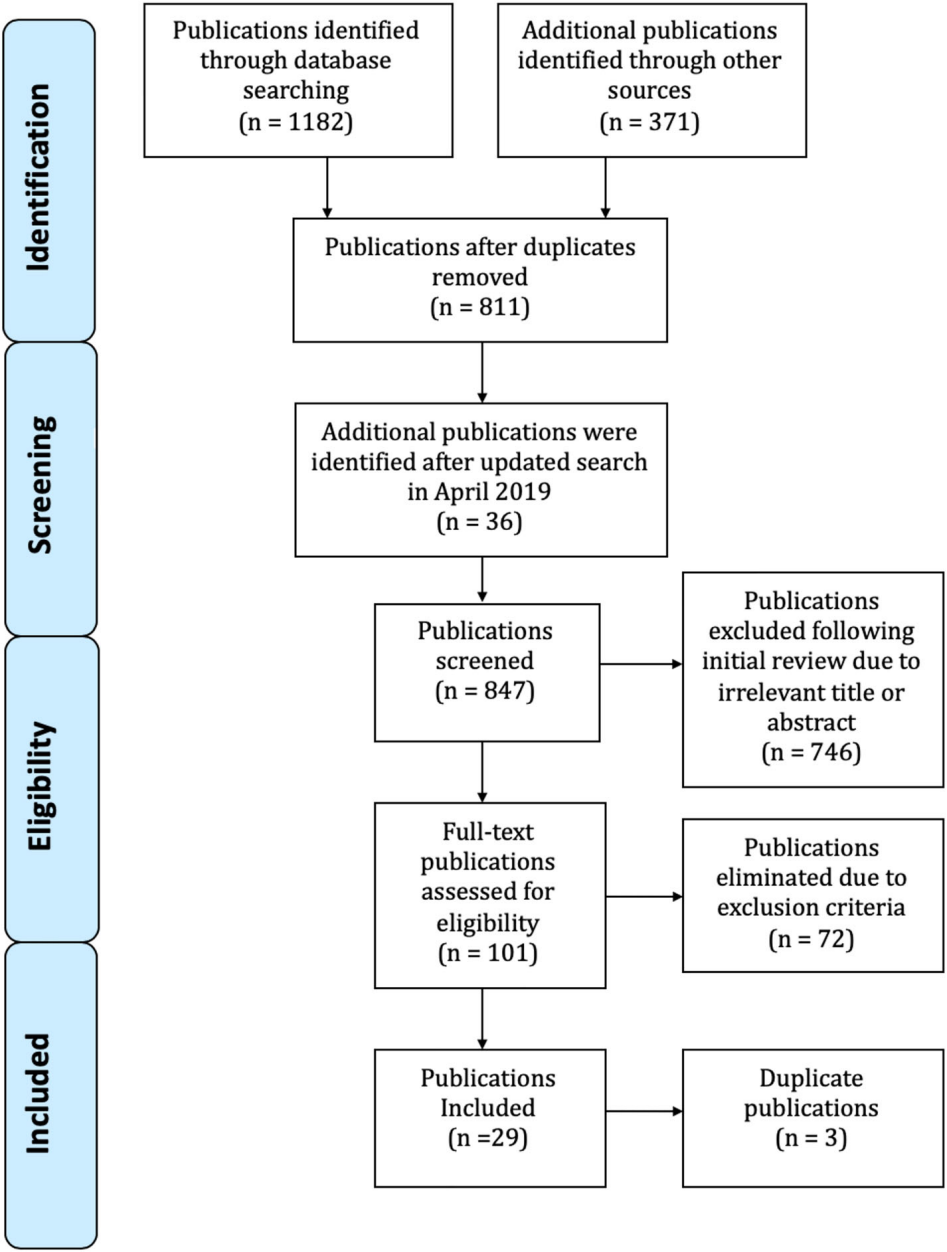
Publication selection methods

One‐hundred one full‐text articles were further assessed for eligibility by the same two reviewers; any conflicts were resolved by a third reviewer (R. C.). A total of 72 articles were excluded for the following reasons: no full‐text available (three papers), not in English at the time of manual review (34 papers), not associated with a periodontal outcome or thyroid outcome (26 papers), related to hyperthyroidism or thyroid cancer but not hypothyroidism (two papers), incorrect/irrelevant comparator (i.e., no comparison between periodontitis and hypothyroidism; two papers), an incorrect or irrelevant intervention (i.e., an intervention not related to periodontal diagnosis; two papers), incorrect/irrelevant patient population (i.e., no comparison of periodontitis and or hypothyroidism; one paper), and if the journals of publication were cited in an open access checklist for predatory publishers (two papers). In addition, three papers were excluded due to duplication. As a result, a total of 28 full‐length papers and one abstract were included for review. The selection process used in our analysis is described in more detail in Figure 1.

## 3 RESULTS

### 3.1 Aim 1

To evaluate the quality and quantity of the existing literature describing the potential relationship between hypothyroidism and periodontitis.

#### 3.1.1 Outcome of search

After applying the inclusion, exclusion, and eligibility criteria noted above, a total of 29 publications, including one abstract, were considered for further analysis as shown in Table 3. We found that published papers describing the relationship between hypothyroid disease and periodontitis generally ranked poorly in the hierarchy of scientific evidence, and most were associated with significant methodological limitations. There were no published meta‐analyses or systematic reviews available that described the thyroid–periodontal disease relationship. Similarly, there were no randomized controlled human clinical trials and only one retrospective cohort study (that investigated dental implants). Only one intervention study and three case–control studies were available. There were 14 cross‐sectional studies (five of them are retrospective chart reviews): two review papers, three case reports, and four animal studies, in addition to one abstract.

**Table 3:** Aim 1: Summary of publications in initial search

Author	Year of publication	Type of study	Association with hypothyroidism	Included/excluded
Alsaadi et al.	2007	Retrospective chart review (cross‐sectional)	No association between hypothyroidism and early implant failure	Excluded
Attard & Zarb	2002	Retrospective chart review (cross‐sectional)	• No association between hypothyroidism and implant failure • Positive association between hypothyroidism and bone loss	Included
Chatzopoulos et al.	2016	Cross‐sectional	No association between hypothyroidism and CPITN scores 3&4	Excluded
Chrysanthakopoulos et al.	2013	Prospective cross‐sectional	Positive association between hypothyroidism and PD > 5 mm	Excluded
Chrysanthakopolulos et al.	2016	Prospective cross‐sectional	Positive association between hypothyroidism and PD > 5 mm	Excluded
Dalago et al.	2017	Cross‐sectional	No association between hypothyroidism and peri‐implantitis	Excluded
Molloy et al.	2004	Retrospective chart review (cross‐sectional)	• Positive association between hypothyroidism and mild bone loss, but not severe • No association with tooth loss	Excluded
Nesse et al.	2010	Cross‐sectional	No association between hypothyroidism and PD	Excluded
Rahangdale et al.	2018	Cross‐sectional	Positive association between hypothyroidism and PD, CAL	Included
Soomsawasdi (a)	1965	Cross‐sectional	Positive association between hypothyroidism and tooth mobility	Excluded
Soomsawasdi (b)	1965	Cross‐sectional	Positive association between hypothyroidism and missing teeth	Excluded
van Steenberghe et al.	2002	Cross‐sectional	No association between hypothyroidism and implant failure	Excluded
Xie & Ainmo	1999	Retrospective chart review (cross‐sectional)	No association between hypothyroidism and tooth loss	Excluded
Zeigler et al.	2015	Cross‐sectional	Positive association between high TSH levels and PD (5 mm and more)	Excluded
Babu et al.	2016	Case–control	Positive association between hypothyroidism and GI, PI (children)	Excluded
Beriashvili et al.	2016	Case–control	Positive association	Excluded
Saima et al.	2016	Case–control	Positive association between hypothyroidism and GI, PI (children)	Excluded
de Souza et al.	2013	Cohort study	No association between hypothyroidism and peri‐implantitis	Excluded
Bhankhar et al.	2017	Interventional	Positive association between hypothyroidism and CAL around 5 mm	Excluded
Patil et al.	2011	Review	Positive association	Excluded
Zahid et al.	2011	Review	Positive association	Excluded
Yerke et al.	2019	Abstract	Positive association	Included
Gupta et al.	2014	Case report	Unknown	Excluded
Patil et al.	2012	Case report	Positive association	Excluded
Yussif et al.	2017	Case report	Positive association	Excluded
De Toledo et al.	1979	Animal study—marmos model	Positive association	Excluded
Feitosa et al.	2009	Animal study—rat model	Positive association	Included
Pinto	1974	Animal study—rat model	Positive association	Excluded
Schneider	1969	Animal study—rat model	No association	Excluded

Abbreviations: BOP, bleeding on probing; CAL, clinical attachment loss; CPITN, the community periodontal index of treatment needs; GI, gingival index; PD, pocket depth; TSH, thyroid‐stimulating hormone.

### 3.2 Aim 2

To assess the relationship between periodontal disease severity and hypothyroidism.

#### 3.2.1 Relationship between hypothyroidism and periodontitis

All 29 publications noted in Table 3 were critically assessed for their ability to address a potential relationship between hypothyroidism and periodontitis. In general, papers were excluded if the patient populations consisted of only children or adolescents, because those groups are typically associated with a low prevalence of hypothyroid or periodontal diseases; if the study investigated primarily gingivitis or included patients having minimal probing depths rather than periodontitis; if there was no control group or baseline data available for data comparison; if potentially unreliable surrogate measurements of periodontitis were used (e.g., tooth loss and implant failure prior to abutment connection); if obsolete diagnostic methods for thyroid disease were used (e.g., measurement of the Achilles tendon reflex as a surrogate for hypothyroidism); reports on histological evaluation of inflammation without measurement of clinical periodontal parameters or bone levels; and studies that used patients with “thyroid disease” without distinguishing among subjects with hypothyroidism, hyperthyroidism, and thyroid cancer.

#### 3.2.2 Papers excluded from analysis of hypothyroid–periodontitis relationships: Rationale and description

Two articles from Table 3 were review papers (Patil, Patil, & Gururaj, 2011; Zahid, Wang, & Cohen, 2011) that referred to primary articles also noted in that table. Consequently, those were excluded from the critical analysis, and the component papers were considered separately.

All the three case–control studies by Beriashvili, Nikolaishvili, Mantskava, Momtsemlidze, and Franchuk (2016), Saima, Tasneem, and Gowhar (2016), and Venkatesh Babu and Patel (2016) were excluded because their subject populations consisted of only children or adolescents. In addition, periodontal measurements in the excluded studies were based solely on the presence of gingival inflammation (gingivitis), and there were no measurements of pocket depth or bone levels. The study by Zeigler, Wondimu, Marcus, and Modéer (2015) also was excluded due to use of an adolescent patient population and a definition of pocket depths greater than 3 mm as “deep.” In addition, the objective of Zeigler et al. was to compare blood pressure to obesity, but the level of thyroid‐stimulating hormone (TSH) was used only as an adjusted confounder for that comparison. Consequently, TSH levels could not be directly assessed to measure the effect of hypothyroidism on periodontal disease.

The intervention study by Bhankhar, Hungund, Kambalyal, Singh, and Jain (2017) also was excluded. Although they found that alveolar bone loss was greater in hypothyroidism patients compared with the healthy controls, one of the limitations of this study was the absence of a control group consisting of normal thyroid function in patients with periodontitis. An additional weakness was that TSH levels were not measured posttreatment in the nonhypothyroidism group, so the posttreatment TSH levels in hypothyroidism group had no direct control comparison.

Two older papers by Soomsawasdi, Ringsdorf, and Cheraskin 1965a, 1965b) were excluded due to the use of a questionable (and currently abandoned) technique for the diagnosis of thyroid disease: the Burdick photomograph and electrocardiograph, which essentially is a measurement of a timed graphic curve of the Achilles tendon reflex for thyroid disease diagnosis. In addition, the authors acknowledged the considerable variability and uncertain appropriateness of their diagnostic methods, the subject population was limited (only females were included), and patient thyroid diagnoses were not subsequently confirmed by medical evaluation or laboratory testing.

The Xie and Ainamo (1999) study was excluded from analysis because their population consisted of only three groups of elderly patients (76, 81, and 86 years old) and hypothyroid disease was not separately reported. More specifically, patients with hypothyroidism, hyperthyroidism, and thyroid cancer were used for their analysis. Dental status was described as either edentulous or dentate (which was defined as having at least one tooth), which might not be appropriate for diagnosis of periodontitis, particularly in their elderly populations, and because no association between diabetes and tooth loss was found when this association has been established. Tooth loss also might be related to nonperiodontal etiologies such as trauma or caries. Finally, the authors also acknowledged that many patients with systemic diseases such as diabetes might not have survived to old age, which is an additional study limitation.

Chatzopoulos and Tsalikis (2016) were excluded because a community periodontal index of treatment needs score of 3 was used for diagnosis of periodontitis, which would include patients having any teeth with pocket depths greater than 3 mm. No additional periodontal criteria (e.g., bone levels and histology) were considered. Moreover, patient populations with hypothyroidism, hyperthyroidism, and thyroid cancer were pooled and used for analysis. In this study, the authors also did not find a relationship between diabetes and periodontitis, which also suggests that their inclusion criteria included a considerable number of patients having gingivitis or only slight periodontitis.

The study by Molloy, Wolff, Lopez‐Guzman, and Hodges (2004) found that thyroid disease was more prevalent among patients with mild bone loss, but not with tooth loss. In their analysis, the authors did not differentiate between hyperthyroidism, hypothyroidism, or thyroid cancer, which may have affected the results. In addition, it is arguable whether measurement of tooth loss is a sufficiently sensitive diagnostic indicator for periodontitis and was therefore excluded from critical analysis.

In the two publications by Chrysanthakopoulos and Chrysanthakopoulos (2013, 2016), a positive association was found between periodontal disease and hypothyroidism. They defined periodontitis by the presence of pocket depths equal or greater than 5 mm and excluded patients that had been recently treated for periodontal disease, to obtain patients with “clinically established periodontitis.” However, those studies also pooled patients with thyroid disease to include hypothyroidism, hyperthyroidism, and thyroid cancer in one category. The authors further reported that as many as one third of their patient pool was reported to have hyperthyroidism. The authors also did not specifically indicate if the endocrine results had been adjusted for diabetes or smoking, and therefore, both studies were excluded.

Nesse et al. (2010) evaluated periodontitis and hypothyroidism in control patients, periodontitis patients in dental clinics, and periodontitis patients in periodontal clinics. The results were adjusted for age, gender, and smoking. However, periodontitis was defined as having pocket depths greater than 3 mm and, although full mouth probing depths were recorded for patients with community periodontal index of treatment needs scores 3 or more, clinical periodontal data were unavailable in 28% of patients, smoking data were missing from 50% of one of the clinic sites, and smoking status was significantly different between the groups. An additional limitation of this study was that patients with periodontitis were significantly older than the control group and more likely to be female, which also might have affected the study outcome.

All of the three case reports by Patil and Giri (2012), Gupta, Goel, Solanki, and Gupta (2014), and Yussif, El‐Mahdi, and Wagih (2017) were excluded because a periodontal diagnosis was not obtained in the Gupta et al. report, there were no periodontal measurements, and the patient's chief complaint was the presence of retained deciduous teeth. The other two case reports (Patil & Giri, 2012; Yussif et al., 2017) were excluded in our analysis, as case reports do not rank highly in the hierarchy of scientific evidence. In both of those cases, there was a periodontal diagnosis based on clinical periodontal measurements such as pocket depth, bleeding on probing, tooth mobility, and radiographic bone loss. In both cases, resolution of periodontal disease was observed following diagnosis and treatment of hypothyroid disease concurrent with periodontal therapy. However, the absence of a control group and the study design remains the major limitation of those papers.

Five papers were related to dental implants and thyroid disease. Of those, four were excluded: van Steenberghe, Jacobs, Desnyder, Maffei, and Quirynen (2002) and Alsaadi, Quirynen, Komarek, and van Steenberghe (2007) were not further considered because they evaluated the relationship between thyroid disease and early implant failure, which included the time period from fixture placement up to—but before—abutment connection. Consequently, implant failure during that period most likely represents a lack of osseointegration and might not be an appropriate indicator of chronic periodontal disease. de Souza et al. (2013) were excluded because measurements were nonstandardized, uncorrected for distortion, and not adjusted using proportionality or computer‐based methods for differences in angulation, which may affect outcomes. de Souza et al., as well as Dalago, Schuldt Filho, Rodrigues, Renvert, and Bianchini (2017), did not find a relationship between peri‐implantitis and smoking, or with peri‐implantitis and diabetes. There also was no distinction among hypothyroidism, hyperthyroidism, and other thyroid disease in either study, because patients with any or all of those conditions were pooled and considered to have “thyroid disease.” Additionally, de Souza et al. also acknowledged that their small sample size might have influenced their results, and only 7.3% of patients in the study by Dalago et al. had a diagnosis consistent with peri‐implantitis.

Our review also identified a number of animal studies relevant to the search terms. Three of those papers were excluded for several reasons. The 1969 rat study by Schneider was not further considered because experimental periodontal disease was not induced in this model, which evaluated “naturally occurring periodontal disease.” In addition, only gingival inflammation was assessed, as well as the presence of “degenerative periodontosis.” There was no control group, and no measurements of bone loss were recorded. Similarly, the rat study by Pinto (1974) and the De Toledo, Bozzo, Do Nascimento, and Sallum (1979) study in marmosets were based on descriptive histology that qualitatively assessed inflammation in the periodontal ligament, without any defined or direct measurements of periodontal disease such as bone loss, pocket depth, or attachment loss. Moreover, no control groups were presented in either of those studies.

#### 3.2.3 Papers included in the analysis of hypothyroid–periodontitis relationships: Rationale and description

Of the 29 publications obtained from the results of the primary search, 25 were excluded as described above and four remained for further descriptive analysis to address Aim 2. The four remaining publications are listed in Table 4 and are described in more detail as follows:

**Table 4:** Aim 2: [Appendix 1] Summary of the publications included for analysis of the periodontitis–hypothyroidism relationship

Author	Year of publication	Type of study	Association between hypothyroidism and periodontitis	Periodontal diagnosis as defined in the paper	Hypothyroid disease as defined in the paper
Attard & Zarb	2002	Retrospective chart review (cross‐sectional)	• No association between hypothyroidism and implant failure • Positive association between hypothyroidism and bone loss (compare with control group)	Radiographic bone loss around implant	• Diagnosed with primary hypothyroidism • On thyroid replacement medication
Feitosa et al.	2009	Animal study (rat model)	Positive association in hypothyroidism rats and bone loss in ligature‐induced periodontitis model	Bone resorption	• Hypo‐thyroidism induced by the ingestion of a solution containing propylthiouracil (Propilracil, 100 mg) • Taking antithyroid drug that blocks thyroid hormone synthesis
Rahangdale et al.	2018	Cross‐sectional	Positive association between hypothyroidism and PD, CAL	Pocket depth, clinical attachment loss, and bone loss	• Diagnosed with primary hypothyroidism On thyroid replacement medication
Yerke et al.	2019	Abstract	Positive association with deep pocket ≥5 or ≥6 mm	Pocket depth	Patient medical history for use of prescription medication(s) for thyroid hormone supplementation

Rahangdale and Galgali (2018), in a comparative cross‐sectional study involving 102 human subjects, found significantly greater mean pocket depths and more clinical attachment loss among patients with hypothyroidism, compared with patients without thyroid disease. Groups were statistically adjusted for age, and plaque scores for both groups were similar. The authors concluded that patients taking thyroxine replacement medications were more likely to have periodontal disease than euthyroid control subjects.

Attard and Zarb (2002) could not demonstrate an association between hypothyroidism and implant *failure*, but there was a relationship between peri‐implant radiographic *bone loss* in patients with hypothyroidism, compared with normal controls. The lack of association with implant failure might be related to the relatively large amount of bone loss required for implant failure. The strengths of this study included patient matching for age, gender, site, and prosthetics between the hypothyroid and control groups, the use of only hypothyroid patients, and use of standardized radiographs. Limitations included small sample size (56 patients), the retrospective nature of their study, and use of self‐reported hypothyroidism in lieu of a formal diagnosis.

One well‐designed animal study by Feitosa et al. (2009) was included in our analysis. The authors used an experimental periodontitis ligature model in a rat model system. Three groups were included in the study: control, hypothyroidism, and hyperthyroidism, and thyroid status was confirmed using laboratory blood testing for TSH. Periodontal bone levels were measured via histomorphometric analysis. They found that an increase in periodontitis‐related bone loss was associated with hypothyroidism (but not with hyperthyroidism).

One abstract by Yerke, Levine, and Cohen (2019) was included in our analysis. That study evaluated the association between hypothyroidism and severity of periodontitis among 538 patients with moderate to severe periodontitis. The extent of periodontal disease was evaluated by measuring the percentage of teeth with probing depths greater than or equal to 5 or 6 mm, and hypothyroidism was assessed through review of patient medical histories for use of prescription thyroid hormone supplementation in combination with a diagnosis of hypothyroidism. They found that patients with hypothyroidism had significantly more teeth with deeper periodontal probing depths, compared with patients without thyroid disease. In that study, all patients were examined by single examiner, but the retrospective natural of that analysis was a limitation because the dosage and duration of thyroid hormone supplementation was not determined.

## 4 DISCUSSION

Our results indicate that there are very few high‐quality studies describing the potential association between hypothyroidism and periodontitis. It appears that most of the available literature consists of retrospective cross‐sectional studies, case reports, and retrospective chart review analyses. Our results are similar to the two review papers (Patil et al., 2011; Zahid et al., 2011) that were identified in our scoping review for Aim 1, both of which found that, although there is evidence that a potential relationship might exist between hypothyroidism and periodontitis, existing literature lacks adequate randomized controlled clinical trials and observational studies to support this claim. Nevertheless, our review suggests that a positive relationship between hypothyroidism and periodontitis most likely exists and that this relationship might be more apparent among subjects with severe periodontitis, compared with patients with mild periodontal disease.

In the critical analysis section of our review, we identified three full‐length papers and one abstract that demonstrated a positive relationship between those two conditions. All the excluded publications were compromised due to lack of defined periodontal measurements, as well as the use of relatively unreliable methods for diagnosis of hypothyroid disease. Consequently, it was not possible to perform a systematic review or meta‐analysis of the periodontitis–thyroid disease relationship. Therefore, a scoping review was performed to assess the quality of the existing literature.

The lack of relevant papers might be due to the effects of thyroid hormone replacement therapy on hypothyroidism, which might mask the effect of untreated hypothyroid disease on periodontal disease, particularly in patients with mild to moderate periodontitis. Indeed, many of the papers considered for review used a variety of definitions for periodontitis, and there was no consistent classification of disease. Due to this inconsistency in disease classification among papers, comparison of results from individual publications was challenging. Furthermore, no high‐level evidence was found upon our search, as no meta‐analysis, systematic reviews, or randomized clinical trials were found. As a result, our analysis was focused on a limited number of cross‐sectional studies and case reports, which have a lower level of evidence due to the retrospective nature of the study design, limited sample size, and a higher potential of bias. Indeed, in the review of periodontal manifestations of systemic diseases from the 2017 World Workshop on the Classification of Periodontal and Peri‐Implant Diseases and Conditions (Albandar, Susin, & Hughes, 2018), an established relationship between hypothyroidism and periodontitis was not described. This omission most likely was due to the lack of relevant literature supporting such an association.

One limitation of our study is that only papers written in English were included in our review, and other studies reporting results in languages other than English might be available.

With those limitations, we believe that there is evidence suggesting that a relationship between hypothyroidism and periodontitis likely exists, and we speculate that the relationship may be more apparent in individuals with more severe periodontitis. Perhaps the strongest support for that conclusion comes from the results of Feitosa et al. (2009), where an experimental model of ligature‐induced periodontitis was evaluated in rats with and without thyroid disease, Rahangdale and Galgali (2018), who found a higher pocket depth and clinical attachment loss in hypothyroid patients compared with healthy patients, and by Yerke et al. (2019), who demonstrated a relationship between those conditions in patients with moderate to advanced periodontitis, as well as by Attard and Zarb (2002), who described an association between hypothyroidism and peri‐implantitis.

Hypothyroid patients appear to have a decreased bone turnover rate secondary to slower resorption during bone remodeling (Mosekilde & Melsen, 1978; Mosekilde, Melsen, Bagger, Myhre‐Jensen, & Schwartz Sorensen, 1977) that results in an increase in bone mass (Eriksen, Mosekilde, & Melsen, 1986). Because thyroid hormone receptors are present in human bone (Abu, Bord, Horner, Chatterjee, & Compston, 1997), it has been proposed that thyroid hormone might act directly on bone cells via specific nuclear receptors or indirectly via increasing the secretion of growth hormone and insulin‐like growth factor (Abu et al., 1997). Patients with hypothyroidism tend to exhibit higher than normal bone density, whereas subjects with hyperthyroidism, characterized by lower levels of TSH, appear to experience bone loss and have a higher fracture incidence (Lakatos, 2003; Mosekilde, Eriksen, & Charles, 1990). Animal models of hypothyroidism also have demonstrated alterations in bone metabolism (Britto, Fenton, Holloway, & Nicholson, 1994; Monfoulet et al., 2011), possibly through a mechanism by which thyroid hormone has direct or indirect effects on osteoblasts and osteoclasts.

Other drugs can affect thyroid function in addition to medications, such as levothyroxine and liothyronine, which are primarily used to treat hypothyroidism. Tyrosine kinase inhibitors used in cancer treatment have the potential to modify thyroid hormone metabolism or directly induce hypothyroidism (Barbesino, 2010; Desai et al., 2006; Mannavola et al., 2007). Immunomodulating agents such as alemtuzumab, interferon, and other cytokines also can affect thyroid function, although the mechanisms have not been fully elucidated (Coles et al., 1999; Eskes & Wiersinga, 2009; Investigators, 2008; Rao, Kremenevskaja, Resch, & Brabant, 2005; Schou, Amdisen, Jensen, & Olsen, 1968; Tran, Malcolm Reeves, Gibson, & Attia, 2009). Other medications known to have thyroid effects include lithium, amiodarone, and oral estrogens (Eskes & Wiersinga, 2009; Rao et al., 2005; Schou et al., 1968; Tahboub & Arafah, 2009).

Our findings also have applicability to clinical practice. Specifically, periodontists should consider thyroid disease, and endocrinologists should consider the presence of periodontitis, during patient evaluation because early diagnosis and intervention for both conditions is likely to mutually improve patient outcomes. Ideally, dentists should be able to recognize local and systemic symptoms that may indicate undiagnosed thyroid diseases, or signs of medications that affect thyroid function, and recommend appropriate patient referrals when necessary. Similarly, physicians should consider clinical history or signs of periodontal diseases during medical evaluations.

From the authors' knowledge, this study is the first scoping review to evaluate the available literature regarding the association between periodontitis and hypothyroidism. Our study also identifies gaps and opportunities for future research in this area by conducting a well‐designed randomized clinical trial that include comprehensive assessment of immune status. A better understanding of the potential effect of thyroid hormone dysfunction in the outcome and severity of periodontitis might be important for the decrease in morbidity of both diseases.

## 5 CONCLUSIONS

There are very few high‐quality studies describing the potential association between periodontitis and hypothyroidism, with most of the available literature consisting of retrospective cross‐sectional studies and chart reviews. In general, a positive association between periodontitis and hypothyroidism was found. Further well‐controlled, prospective clinical and immunologic studies will be required to confirm that relationship, to measure the strength of any association with disease severity, to establish causality, and to establish the role of either disease in the pathogenesis of the other.

## CONFLICT OF INTEREST

None of the authors have any financial or commercial associations or conflicts of interest.

## CLINICAL RELEVANCE

## SCIENTIFIC RATIONALE FOR THE STUDY

The potential association between periodontitis and a variety of immunologic and inflammatory conditions provides a foundation for a possible relationship between hypothyroidism and periodontitis.

## PRINCIPAL FINDINGS

There are very few high‐quality studies describing the potential association between hypothyroidism and periodontitis. Nevertheless, analysis of the most relevant clinical and experimental studies supported the existence of a positive relationship between hypothyroidism and periodontitis.

## PRACTICAL IMPLICATIONS

Periodontists should consider thyroid disease, and endocrinologists should consider the presence of periodontitis, during patient evaluation because early diagnosis and intervention for both conditions is likely to mutually improve patient outcomes.
